# Factors influencing early obliteration during flow diverter treatment of cerebral aneurysms: Establishment of an early obliteration inhibition score

**DOI:** 10.20407/fmj.2022-033

**Published:** 2023-05-09

**Authors:** Akiko Hasebe, Ichiro Nakahara, Shoji Matsumoto, Jun Morioka, Jun Tanabe, Sadayoshi Watanabe, Kenichiro Suyama, Takuma Ishihara, Yuichi Hirose

**Affiliations:** 1 Department of Comprehensive Strokology, Fujita Health University, School of Medicine, Toyoake, Aichi, Japan; 2 Innovative and Clinical Research Promotion Center, Gifu University Hospital, Gifu, Gifu, Japan; 3 Department of Neurosurgery, Fujita Health University, Graduate School of Medicine, Toyoake, Aichi, Japan

**Keywords:** Cerebral aneurysm, Flow diverter, Prognosis, Early obliteration inhibition, Scoring system

## Abstract

**Objective:**

This retrospective study aimed to investigate factors associated with inhibition of early aneurysm obliteration after flow diverter (FD) treatment. We also created the early obliteration inhibition (EOI) score for pre-operative evaluation.

**Methods:**

We examined 110 cerebral aneurysms in 104 patients who underwent FD treatment. The following parameters were investigated: age, sex, symptoms, aneurysm location and type, maximum aneurysm diameter, parent vessel diameter, neck diameter, and dome–neck ratio. We also noted aneurysm location relative to the curvature of the parent artery and any branches arising from the aneurysm dome. Procedural factors such as FD diameter and length, number of FDs placed, type of FD, and use of adjunctive coiling were also investigated. Aneurysm obliteration was evaluated using digital subtraction angiography 3 months after the procedure. Adequate obliteration was defined as grade C or D on the O’Kelly–Marotta scale.

**Results:**

The following factors inhibited early obliteration: 1) extradural location, 2) saccular aneurysm, 3) aneurysm neck located at the outer convexity of the parent artery, and 4) arterial branch arising from the aneurysm dome. Odds ratios were used to create an EOI score. Receiver operating characteristic curve analysis showed that the optimal cut-off EOI score for adequate obliteration was 1.5 (area under the curve, 0.81; 95% confidence interval, 0.73–0.9; sensitivity, 0.9; specificity, 0.57).

**Conclusion:**

The EOI score, which is based on factors that inhibit early obliteration, may predict early treatment outcomes of FD placement.

## Introduction

In recent years, remarkable progress has been made in endovascular treatment of cerebral aneurysms. More than half of unruptured cerebral aneurysms treated in Japan are treated using an endovascular approach. Although conventional endovascular treatment involves endosaccular embolization using detachable coils, the introduction of flow diverter (FD) placement for side-wall type unruptured aneurysms has caused a paradigm shift in treatment.^1^ The mechanism of aneurysm obliteration with flow diversion has been described in previous studies. Briefly, the FD is placed to cover the aneurysm neck, which promotes thrombosis within the aneurysm and provides a scaffold for endothelial cell growth along the device.^1,2^ The Pipeline embolization device (Medtronic, Minneapolis, MN, USA) was the first FD approved for use. The Pipeline Flex (Medtronic) is an improved version that was introduced in Japan in 2015 for use in wide-necked proximal internal carotid artery aneurysms with a major diameter of at least 10 mm.^3^ In 2020, the Pipeline Shield (Medtronic), which has improved operability and antithrombotic properties, was introduced for use in internal carotid and vertebral artery aneurysms with a major diameter of at least 5 mm.^4–6^ Another new FD, the Flow Redirection Endoluminal Device (FRED; MicroVention-Terumo, Aliso Viejo, CA, USA), is now indicated for use in aneurysms of the proximal anterior and middle cerebral arteries and basilar artery.^7–9^ Therefore, a considerable number of cerebral aneurysms can now be treated using a FD.

The reported rates of complete aneurysm obliteration 1 year after FD treatment range from 70% to 80%.^1,4–11^ Early obliteration prevents aneurysmal rupture and enlargement and enables dose reduction and termination of antiplatelet therapy.^12^ This study aimed to elucidate factors that inhibit early obliteration at the three-month stage after FD treatment and establish an early obliteration inhibition (EOI) score to identify patients and aneurysms less suitable for FD treatment.

## Materials and Methods

Patients with unruptured cerebral aneurysms who successfully underwent FD placement and subsequent imaging follow-up three months after treatment for five years since October 2016, which was when FD was introduced at our hospital, were eligible for study inclusion. We excluded patients under age 18 years and those who had undergone previous coil embolization or FD placement. Patients who required additional treatment within 3 months of FD placement were also excluded. Clinical and radiological data were obtained from a retrospective review of the medical records and neuroimaging studies. All patients provided written informed consent for treatment. The study received institutional review board approval. The requirement for informed consent for study participation was waived owing to the retrospective nature of the study and because all data were de-identified.

### Peri-operative antiplatelet therapy

Dual antiplatelet therapy (aspirin 100 mg/day and clopidogrel 75 mg/day) was initiated in all patients 2 weeks before treatment. Platelet function was evaluated using VerifyNow (Accumetrics, San Diego, CA, USA) 2 days before treatment.^12^ In patients with >550 aspirin reaction units (ARUs), aspirin dosage was increased to 200 mg/day. In patients with ≥210 P2Y12 reaction units (PRUs), clopidogrel was stopped and a loading dose of prasugrel 20 mg was administered followed by 3.75 mg/day. In patients with 60–210 PRUs, clopidogrel 75 mg/day or prasugrel 3.75 mg/day was administered. In patients with <60 PRUs, the prasugrel dosage was reduced to 1.88 mg/day. Repeat platelet function testing was performed again before hospital discharge; dosage changes were made accordingly.^12^

### Overview of standard treatment technique

FDs were placed with the patient under general anesthesia. An 8Fr sheath introducer was placed into the superficial femoral artery, followed by administration of 70–100 U/kg of unfractionated heparin. Heparin was titrated to maintain activated clotting time (ACT) >250 seconds. ACT was measured hourly.

An 8Fr guiding catheter (ROADMASTER, GOODMAN, Aichi, Japan) was advanced into the parent vessel of the target aneurysm. A 5Fr Navien 115 cm (Medtronic) or 5Fr Sofia Select (MicroVention-Terumo) catheter was used as an intermediate catheter. The Marksman (Medtronic) or Phenom 27 (Medtronic) microcatheters were used for placement of the Pipeline Flex or Pipeline Shield. The Headway Plus 27 (MicroVention-Terumo) microcatheter was used to place the FRED. FDs were guided to the aneurysm through the intermediate catheter. FDs were selected at the operator’s discretion according to the course of the parent artery.^8^ The parent artery diameter was measured at the distal, neck, and proximal positions of the aneurysm. FD size was determined based on the maximum diameter of the parent artery and the length of the aneurysm neck. The FD was guided across the neck of the aneurysm and deployed. Percutaneous transluminal angioplasty was performed if high-resolution cone-beam computed tomography depicted insufficient adaptation of the FD against the parent arterial wall. Endovascular coiling was performed in intradural aneurysms with major diameter ≥10 mm if a bleb or jet blood flow into the aneurysm was observed. Brain magnetic resonance imaging was performed the day after treatment. Patients were discharged home approximately 7 days after the procedure.

### Follow-up imaging and antiplatelet therapy

Digital subtraction angiography (DSA) was performed 3, 6, and 12 months after the procedure. Dual antiplatelet therapy was continued for at least 6 months. and the DSA results were used to reduce the number of drugs to one at the 6–12-month mark and discontinue therapy at the 18–24-month mark. Since the purpose of this study was to evaluate patients after 3 months of treatment, all included patients were on DSA and on dual antiplatelet drugs at the time of evaluation.

### Study parameters

The following patient and aneurysm characteristics were recorded: age, sex, symptoms, aneurysm location and type, maximum aneurysm diameter, parent vessel diameter, neck diameter, and dome–neck ratio. We also noted aneurysm location relative to the curvature of the parent artery and any branches arising from the aneurysm dome. Procedural factors such as FD diameter and length, number of FDs used, type of FD, and use of adjunctive coiling were also recorded. Aneurysm obliteration was evaluated using the O’Kelly–Marotta (OKM) grading scale^13^: A, at least 95% of the aneurysm remains; B, 5%–95% remains; C, <5% remains; and D, complete obliteration. Adequate obliteration was defined as OKM grade C or D.

### Statistical analysis

Continuous data are presented as medians with interquartile range (IQR). Categorical data are presented as numbers with percentage. A multivariable ordinal logistic regression model was generated to explore factors associated with adequate obliteration 3 months after the procedure. The following factors were selected *a priori* based on previous studies^2,14–16^ because they are likely to be associated with aneurysm obliteration:

Patient factor: age
Aneurysm factors: intra/extradural location, anterior/posterior circulation location, maximum aneurysm diameter, parent artery diameter, dome–neck ratio, saccular/dissecting aneurysm, aneurysm location relative to the curvature of the parent artery (yes/no) and arterial branch arising from the aneurysm dome (yes/no)
Procedural factors: number of FDs, type of FD, and adjunctive coiling

Ordinal logistic regression analysis was performed to calculate odds ratios (ORs) with 95% confidence intervals (CIs) and determine factors associated with early adequate obliteration. Penalized maximum likelihood estimation was used to allow shrinkage of the effect of factors to correct for possible overfitting. Factors associated with inhibition of early obliteration were used to create a binary logistic regression predictive model. A simple score was developed to predict the EOI score based on the regression coefficients of the binary logistic regression model. Receiver operating characteristic (ROC) curves and optimal cut-off points were calculated. Two-sided P<0.05 was considered significant. Statistical analyses were performed using R software version 4.1.1 (R Foundation, Vienna, Austria).

## Results

This study included 110 aneurysms in 104 patients. Among the 134 patients treated during the study period, 30 were excluded based on criteria: one patient in whom FD placement was not successful, two under age 18 years, 23 who underwent FD after a previous procedure, three who were lost to follow-up, and one who underwent additional treatment within 3 months of the FD procedure. Eleven patients (10.5%) experienced an adverse event, including cerebral infarction with residual sequelae (n=3) and carotid–cavernous sinus fistula without sequelae (n=4). All four carotid–cavernous fistulae required additional treatment. In one patient, FRED placement was not successful and a Pipeline Shield had to be placed instead. No deaths occurred.

Patient and aneurysm characteristics are shown in Table 1. The number of aneurysms classified as OKM grade A, B, C, and D on follow-up DSA performed 3 months after treatment was, 12 (10.9%), 19 (17.2%), 16 (14.5%), and 63 (57.3%), respectively. Adequate obliteration (OKM grade C or D) was achieved in 79 aneurysms (71.8%).

In ordinal logistic regression analysis, the following factors were significantly associated with inhibition of early obliteration: extradural location (OR 3.55; 95% CI, 1.3–9.74; P=0.014), saccular aneurysm (OR 4.01; 95% CI, 1.12–14.31; P=0.032), aneurysm neck located at the outer convexity of the parent artery (OR 3.08; 95% CI, 1.31–7.23; P=0.01), and arterial branch arising from aneurysm dome (OR 6.86; 95% CI, 2.12–22.14; P=0.001). Binary logistic regression performed using these four factors showed that extradural location (OR 4.54; 95% CI, 1.55–13.26; P=0.006), saccular aneurysm (OR 4.15; 95% CI, 1.12–15.4; P=0.033), and aneurysm neck located at the outer convexity of the parent artery (OR, 7.04; 95% CI, 2.51–19.78; P<0.001) were significantly associated with inhibition of early obliteration. Although presence of an arterial branch arising from the aneurysm dome failed to reach statistical significance by a small margin (OR, 3.45; 95% CI, 0.9–13.22; P=0.07), we still included it in the EOI score because of strong sign of association in ordinal logistic regression analysis (Table 2).

**Figure 1:**
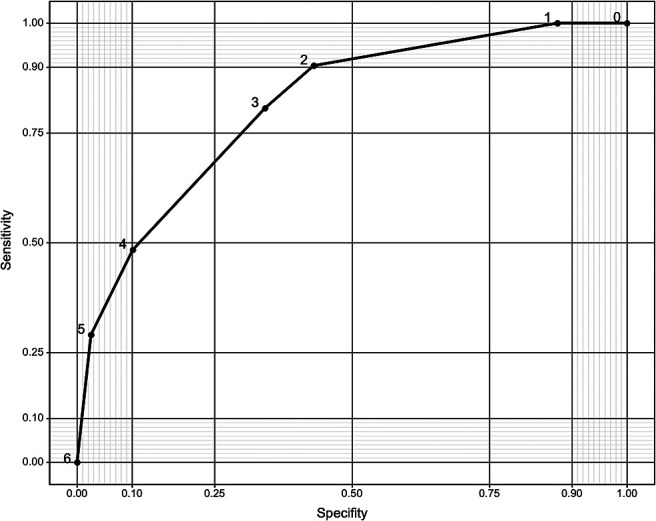
Receiver operating characteristic curve for prediction of adequate obliteration using the early obliteration inhibition score.

The EOI score was created using the regression coefficients from the binary logistic regression model. Scores were weighted by rounding coefficient values: 2 for extradural location and aneurysm neck located at the outer convexity of the parent artery, and 1 for saccular aneurysm and arterial branch arising from the aneurysm dome (Table 3). Figure 1 shows the ROC curve for prediction of adequate obliteration using the EOI score. The optimal cut-off value for adequate obliteration was 1.5 (area under the curve, 0.81; 95% CI, 0.73–0.9; sensitivity, 0.9; specificity, 0.57).

## Representative cases

### Patient 1

**Figure 2:**
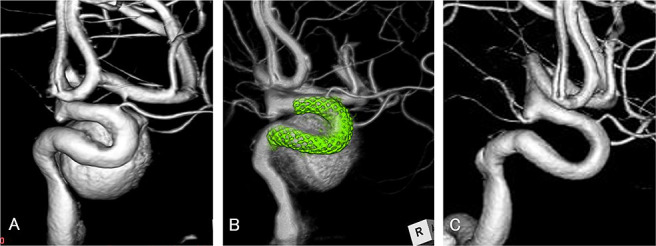
Patient 1 was a 64 year-old woman with a right paraclinoid internal carotid artery aneurysm who presented with right oculomotor palsy. A. Preoperative three-dimensional digital subtraction angiography showed a large aneurysm with 18.5 mm maximum diameter. Preoperative early obliteration inhibition score was 1 (intradural location, 0; saccular aneurysm, 1; aneurysm neck not located at the convexity of the parent artery, 0; no arterial branch arising from aneurysm dome, 0), which suggested early obliteration. B. A Pipeline Shield (diameter, 4.25 mm; length, 16 mm) flow diverter was placed successfully without complication. C. Follow-up angiography 3 months after the procedure showed complete aneurysm obliteration.

A 64-year-old woman presented with a right oculomotor palsy caused by a right paraclinoid internal carotid artery aneurysm. Maximum aneurysm diameter was 18.5 mm and the neck measured 5.5 mm. A Pipeline Shield (diameter, 4.25 mm; length, 16 mm) was placed as described above. Antiplatelet therapy was administered as described above. DSA 3 months after the procedure showed complete aneurysm obliteration. In this case, the preoperative EOI score was 1 (intradural location, 0; saccular aneurysm, 1; aneurysm neck not located at the outer convexity of the parent artery, 0; no arterial branch arising from the dome, 0). We therefore expected early obliteration with FD treatment, which was demonstrated on follow-up DSA (Figure 2).

### Patient 2

**Figure 3:**
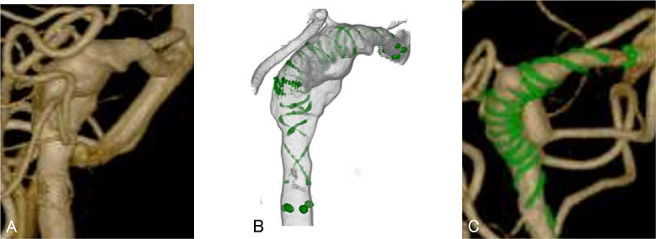
Patient 2 was a 60 year-old man with an incidentally found dissecting aneurysm of the right vertebral artery. A. Pre-operative three-dimensional digital subtraction angiography showed the posterior inferior cerebellar artery arose from the dome. Preoperative early obliteration inhibition score was 3 (intradural location, 0; dissecting aneurysm, 0; aneurysm neck located at the outer convexity of the parent artery, 2; arterial branch arising from aneurysm dome, 1), which suggested early obliteration was unlikely. B. A Flow Redirection Endoluminal Device (diameter, 3.5 mm; effective length, 35 mm) was placed without complication. C. Follow-up angiography 3 months after the procedure showed residual aneurysm.

A 60-year-old man presented with an incidental asymptomatic right vertebral artery aneurysm that exhibited arterial dissection and partial thrombosis of the dome. A FRED (diameter, 3.5 mm; effective length, 35 mm) was placed without complication as described above. Antiplatelet therapy was administered as described above. DSA 3 months after the procedure showed residual aneurysm (OKM grade B). In this case, the preoperative EOI score was 3 (intradural location, 0; dissecting aneurysm, 0; aneurysm neck located at the outer convexity of the parent artery, 2; arterial branch arising from aneurysm dome, 1) and early obliteration was not expected (Figure 3).

## Discussion

Since endovascular coiling of cerebral aneurysms was first introduced in 1997, use of endovascular treatment techniques has increased. FDs, which were initially developed to treat sidewall-type unruptured aneurysms occurring on the lateral wall of the parent artery, have been found safe and effective.^2,3^ FDs currently available for use include the Pipeline Flex, Pipeline Shield, and FRED. Many cerebral aneurysms are amenable to FD treatment.

Numerous large-scale studies have investigated outcomes of FD treatment. The Pipeline for Uncoilable or Failed Aneurysms trial (PUFS trial) evaluated the Pipeline embolization device and reported 6- and 12-month obliteration rates of 74% and 87%, respectively.^10,11^ The 1- and 5-year rates of complete obliteration in a large single-center study of the Pipeline embolization device in Argentina were 75.8% and 96.4%, respectively.^1^ In the Prospective Study on Embolization of Intracranial Aneurysms with the Pipeline device (PREMIER study), the complete obliteration rate at 1 year was 84%.^6^ Two other studies have reported 1-year obliteration rates of 77.2% and 81.8%, respectively.^4,5^ Regarding the FRED, reported 1-year complete obliteration rates range from 62% to 73%.^7,9^

These results show that the aneurysm obliteration rate increases over time; nonetheless, not all aneurysms progress to complete obliteration. In these aneurysms, retreatment is warranted. The ability to predict the need for retreatment at an early stage would be clinically beneficial. Furthermore, dual antiplatelet therapy is required after FD placement; however, early discontinuation may be required in certain patients because of comorbidities.^12^ Therefore, the ability to predict early obliteration can contribute to the pharmacotherapeutic strategy. In our study, adequate obliteration (OKM grade C or D) was achieved 3 months after treatment in 71.8% of aneurysms, which is comparable to previously reported 1-year rates. We believe our study is the first to report early obliteration status at the 3-month timepoint.

The following four factors inhibited early aneurysm obliteration in our study: 1) extradural location, 2) saccular aneurysm (versus dissecting aneurysm), 3) location of the aneurysm neck on the outer convexity of the parent artery, and 4) presence of an arterial branch arising from the aneurysm dome. Subsequently, ORs were used to create an EOI score. In ROC analysis, the optimal EOI score cut-off value for adequate obliteration was 1.5. In other words, an EOI score <1 indicates that early obliteration is likely while a score ≥2 suggests it is not. Our findings may enable clinicians to predict treatment course and perioperative management. Furthermore, they should help determine whether FD treatment is even indicated.

Our study has several limitations. It was retrospective in design and was conducted in a single center. Therefore, the sample size was small and selection bias may have been introduced. In addition, we excluded patients younger than age 18 years, those with previously treated aneurysms, and patients who required retreatment after FD placement.

## Conclusion

We clarified factors that inhibit early obliteration of cerebral aneurysms after FD treatment. The EOI score, which is determined based on these factors, has the potential to predict early obliteration. Future prospective studies are warranted to verify our findings.

## Figures and Tables

**Table1:** Patient and aneurysm characteristics

Variable		Number
*Patient factor*		
Age^§^		58 years (48–70)
Gender	Female	84 (76.4%)
	Male	26 (23.6%)
*Aneurysm factor*		
Side	Left	59 (55.1%)
	Right	48 (44.9%)
	Midline	3 (2.7%)
Parent artery	ICA	91 (82.7%)
	ACA	1 (0.9%)
	MCA	1 (0.9%)
	VA	16 (14.5%)
	BA	1 (0.9%)
Intra/extradural location^§^	Intradural	82 (74.5%)
	Extradural	28 (25.5%)
Circulatory location^§^	Anterior	92 (83.6%)
	Posterior	18 (16.4%)
Maximum aneurysm diameter^§^		7.6 mm (5.9–11.7)
Parent artery diameter^§^		4.5 mm (3.8–5.0)
Neck diameter		5.3 mm (4.0–7.3)
Dome/neck ratio^§^		1.6 (1.2,1.9) ^†^
Type of aneurysm^§^	Dissecting	28 (25.5%)
	Saccular	82 (74.5%)
Aneurysm neck located at the outer convexity of the parent artery^§^		47 (42.7%)
Branch arising from aneurysm dome^§^		16 (14.5%)
Symptomatic^§^		15 (13.6%)
*Procedural factor*		
FD diameter		4.5 mm (4.0–4.8)
FD length		22.0 mm (18.0–30.0)
Number of FDs deployed^§^	One	97 (88.2%)
	Two	13 (11.8%)
Type of FD^§^	Pipeline Flex	23 (20.9%)
	Pipeline Shield	43 (39.1%)
	FRED	44 (40.0%)
Adjunctive coiling^§^		16 (14.5%)

Values shown are medians with interquartile range or numbers with percentage ^§^ Variables for the multivariable ordinal logistic regression model ICA, internal cerebral artery; ACA, anterior cerebral artery; MCA, middle cerebral artery; VA, vertebral artery; BA, basilar artery; FD, flow diverter; FRED, Flow Redirection Endoluminal Device

**Table2:** Early obliteration inhibition factors with statistical significance

Variable	Odds Ratio	95% lower confidence interval	95% upper confidence interval	P-value
Extradural location (vs. intradural location)	4.54	1.55	13.26	0.006
Saccular aneurysm (vs. dissecting aneurysm)	4.15	1.12	15.4	0.033
Aneurysm neck located on the outer convexity of the parent artery	7.04	2.51	19.78	<0.001
Arterial branch arising from aneurysm dome	3.45	0.9	13.22	0.07

**Table3:** Early obliteration inhibition score obtained from the binary logistic regression model

Variable		Coefficient	Score
Intra/extradural location	Extradural	1.5124	2
	Intradural	0	0
Type of aneurysm	Saccular	1.4243	1
	Dissecting	0	0
Aneurysm neck located on the outer convexity of the parent artery	Yes	1.9519	2
	No	0	0
Arterial branch arising from aneurysm dome	Yes	1.2391	1
	No	0	0
